# Quantitative holographic analysis in stallion spermatozoa following cryopreservation

**DOI:** 10.1038/s41598-025-24885-w

**Published:** 2025-12-04

**Authors:** Maria Antonietta Ferrara, Graziano Preziosi, Raffaele Boni, Raffaella Ruggiero, Stefano Cecchini Gualandi

**Affiliations:** 1 Institute of Applied Sciences and Intelligent Systems , Unit of Naples Italian National Research Council (ISASI-CNR) , Via Pietro Castellino 111, 80131 Napoli, Italy; 2 Department of Basic and Applied Sciences (DiSBA), University of Basilicata, Via dell’Ateneo Lucano, 10, 85100 Potenza, Italy

**Keywords:** Holographic tomography, Sperm freezing, Refractive index, Acrosomal integrity, Freezing extender, Horse, Applied physics, Optical physics, Animal physiology

## Abstract

This study employs Holographic tomography (HT) to examine structural and biophysical changes occurring during the cryopreservation of stallion sperm. HT is an advanced imaging technique that integrates digital holography with tomography to achieve three-dimensional, quantitative reconstructions of objects without the need for treatment or reporter dyes. By using refractive index (RI) intervals to represent specific structural regions of sperm cells, variations in optical density, surface area, volume, and dry mass across different cryopreservation extenders and donors have been quantified. Three main sperm components, (i) nuclear region, (ii) post-acrosomal region and midpiece and (iii) whole cell were identified and discriminated based on different RI. Our results revealed significant differences in volume of post-acrosomal region and midpiece among stallions as well as between fresh and frozen/thawed sperm, whereas no significant differences were observed between freezing extenders, aligning with our findings on sperm kinetics. A significant stallion-extender interaction underscores the need to personalize the sperm freezing process.

**Supplementary Information:**

The online version contains supplementary material available at 10.1038/s41598-025-24885-w.

## Introduction

Cryopreservation is currently the only available technique for efficient sperm storage and is widely used to preserve biodiversity, manage genetic resources, and maintain fertility over time. In equines, it plays a crucial role in preserving high-quality genetic material, facilitating long-distance breeding, improving biodiversity and breeding programs, reducing disease transmission risks, and providing access to high-quality stallions at any time^1,2^.

The process of semen cryopreservation involves collecting sperm, diluting it with freezing extenders containing cryoprotectant compounds, and storing it in liquid nitrogen. Cryoprotectants are small, highly permeable molecules that protect sperm cells during freezing and thawing by preventing damage caused by ice crystal formation. These substances lower the freezing point of the sample and minimize ice formation^3^. Cryopreservation halts cellular processes and enables long-term sperm storage, potentially preserving it indefinitely. The freezing process includes an incubation phase in a freezing extender to allow the exchange of permeant cryoprotectant molecules with intracellular water (osmotic equilibrium)^4^. Simultaneously, the temperature is gradually reduced to slow cellular metabolism and mitigate the toxic effects of cryoprotectants until the sample reaches approximately 4 °C. This temperature reduction can conveniently begin during the transport and handling of semen prior to dilution with the freezing extender.

Despite its benefits, the freezing and thawing process inevitably damages cellular structures, such as the plasma membrane, acrosome, and mitochondria. Ice crystal formation significantly reduces cell viability^5,6^, impairing sperm functionality^7^ and diminishing its fertilizing capacity^8^. This highlights the need for optimized cryopreservation protocols that maximize sperm viability and preserve its functionality.

A critical factor influencing post-thaw sperm quality is the use of appropriate extenders^9^. These media provide a supportive environment that maintains cell integrity, supplies nourishment, and minimizes freezing-induced damage. Cryoprotectants within these extenders play a vital role in shielding sperm during cryopreservation^10^.

Although sperm cryopreservation in stallions has become more widespread, it still faces inefficiencies, primarily due to individual variations in stallions’ capacity to produce sperm that withstands cryogenic stress^11,12^. The reasons behind this variability remain unclear, with most studies relying on in vitro methods ranging from basic to advanced techniques to enhance the accuracy of fertility assessments. Oxidative stress during freezing is believed to contribute to this variability^13^; however, it remains unknown why some stallions’ semen is more affected by oxidative stress than others. Employing differentiated analytical methods could provide valuable insights into addressing these inefficiencies more effectively.

Morphological analysis is a useful tool for assessing sperm quality and detecting freezing-induced alterations^14–16^. However, conventional optical microscopy has limitations due to the transparency of sperm cells and often requires invasive staining techniques that may interfere with cell functionality.

Holographic techniques address these issues by enabling label-free, non-invasive analysis of spermatozoa, providing quantitative insights into their morphology and motility. Digital Holographic Microscopy (DHM) and Quantitative Phase Imaging (QPI) allow detailed characterization of sperm subcellular structures, such as the head, midpiece, and tail. These methods have been applied to both human and animal sperm, offering advantages over traditional staining techniques by preserving cell viability and enabling real-time assessment^17–19^.

Recent studies have demonstrated the potential of holographic approaches for evaluating sperm quality, detecting structural abnormalities, and assessing sperm dynamics in different environmental conditions (e.g., cryopreservation)^20^. Advances in computational techniques, including artificial intelligence and machine learning, have further enhanced the accuracy of holographic sperm analysis, enabling automated classification of sperm subpopulations based on refractive index (RI) properties^21^. Despite these advancements, challenges remain in standardizing protocols and improving resolution for finer structural analysis. However, ongoing research continues to refine these methods, reinforcing their role as valuable tools in reproductive biology and assisted reproductive technologies.

Holographic tomography (HT) has emerged as powerful tool for non-invasive study of sperm structure and integrity^22^. HT enables high-resolution, three-dimensional (3D) imaging of spermatozoa based on RI variations, related to intrinsic physical properties like density and composition, allowing precise visualization of sperm morphology and intracellular structures without the need for labels or dyes. This technique provides quantitative data on cell volume, dry mass, and surface area, offering new possibilities for assessing the impact of cryopreservation on spermatozoa. Moreover, its low phototoxicity, achieved by using low-intensity lasers, and the absence of photobleaching, typically occurring with fluorophores, make it suitable for studying live samples and dynamic processes. The technique’s high-speed image acquisition further facilitates the study of these processes.

In this study, we investigated the effects of different freezing extenders on stallion sperm using HT. By analyzing RI values, we assessed structural changes post-cryopreservation and explored inter-individual variability. Understanding the impact of cryopreservation on sperm morphology and biophysical properties will contribute to the development of optimized preservation strategies tailored to individual stallions. Furthermore, the use of HT allows for a more detailed evaluation of sperm freezability, helping refine semen processing techniques and improving reproductive outcomes in equine breeding programs. Although the holotomographic system used in this study is commercially available, it has been applied in only a few studies on spermatozoa and, to the best of our knowledge, never on stallion spermatozoa. Thus, the novelty of our work lies in the biological insights obtained, as we report for the first time the effects of cryoprotectants and cryopreservation on quantitative biophysical parameters of equine spermatozoa.

## Results

Semen samples were collected from 11 stallions. Each ejaculate was characterized prior to cryopreservation and subsequently divided into four aliquots, which were diluted using four different extenders (Spectrum Duo Red, BotuCrio, INRA Freeze, and HF-20).

General information on age and selected semen traits from ejaculates collected from the eleven stallions are presented in Table 1.

**Table 1:** Mean (± SD) values of age and selected semen traits from ejaculates collected from the eleven stallions included in this study.

		Mean ± SD
Age	years	11.1 ± 4.3
Gel-free semen volume	mL	49.9 ± 22.2
Sperm concentration	x 10^6^	213 ± 125
Total sperm count/ejaculate	x 10^9^	6.38 ± 3.75

### Sperm kinetics

**Fig. 1:**
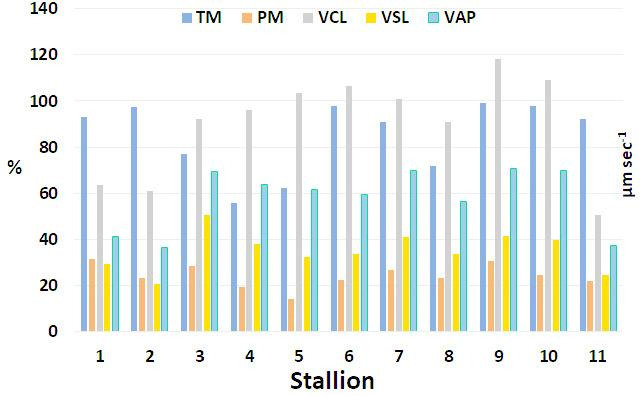
Comparison of Kinematic Parameters in Fresch Stallion Sperm. Kinematic parameters of spermatozoa before freezing, including total motility (TM, %), progressive motility (PM, %), curvilinear velocity (VCL, µm s⁻¹), straight-line velocity (VSL, µm s⁻¹), and average path velocity (VAP, µm s⁻¹), in the eleven stallions enrolled in this study.

**Fig. 2:**
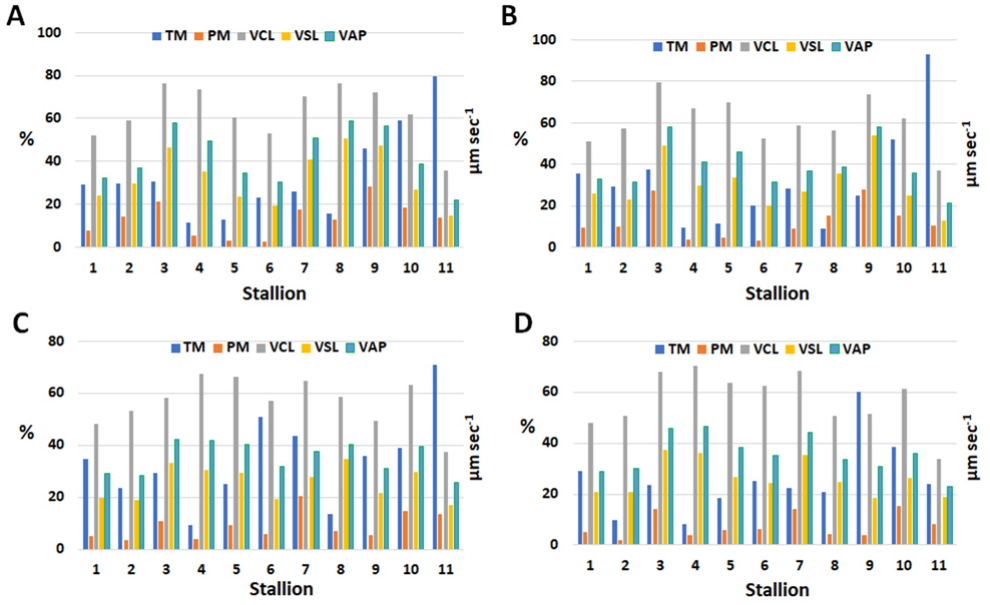
Comparison of Kinematic Parameters Using Different Cryopreservation Extenders. Kinematic parameters of spermatozoa after cryopreservation, including total motility (TM, %), progressive motility (PM, %), curvilinear velocity (VCL, µm s⁻¹), straight-line velocity (VSL, µm s⁻¹), and average path velocity (VAP, µm s⁻¹), in the eleven stallions studied. Each ejaculate was divided and frozen with four extenders: Spectrum Duo Red (**A**), BotuCrio (**B**), INRA Freeze (**C**), and HF-20 (**D**).

The sperm kinematic parameters of ejaculates collected from eleven stallions before cryopreservation are presented in Fig. 1, while Fig. 2 shows the sperm kinetics of the cryopreserved samples obtained using the four extenders under investigation. These results are summarized in Table 2. To evaluate cryotolerance, total motility (TM) and progressive motility (PM) values recorded after freezing were compared with their corresponding pre-freezing values. Based on this comparison, the freezability index was calculated (Figure S1). Analysis of sperm kinematic parameters revealed significant individual variability (*p* < 0.01) across all variables assessed (TM, PM, VCL, VSL, and VAP). A significant decrease (*p* < 0.01) in all parameters was also observed when comparing fresh with frozen spermatozoa, irrespective of the freezing extender used. In contrast, no significant differences were detected among the four freezing protocols/extenders for any parameter. The variable kinematic performance of ejaculates cryopreserved with different extenders suggests that spermatozoa within the same ejaculate can respond differently to freezing, depending on the protocol or extender applied.

**Table 2:** Mean (± SD) values of selected kinematic parameters in spermatozoa diluted with INRA96 prior to cryopreservation, and following cryopreservation with four extenders (Spectrum duo Red, BotuCrio, INRA Freeze, and HF-20) in the eleven stallions enrolled in this study.

Extender		Fresh sperm	Frozen-thawed sperm							
		INRA96	Spectrum Duo Red		BotuCrio		INRA Freeze		HF-20	
		mean ± SD	mean ± SD		mean ± SD		mean ± SD		mean ± SD	
TM	%	85.0 ± 15.5	33.1 ± 20.9		31.8 ± 24.2		34.1 ± 17.3		25.4 ± 14.2	
PM	%	24.2 ± 5.1	13.2 ± 8.0		12.3 ± 8.6		9.0 ± 5.4		7.6 ± 4.7	
VCL	µm s^−1^	90.2 ± 22.0	62.8 ± 12.6		60.4 ± 11.9		56.6 ± 9.2		57.7 ± 11.1	
VSL	µm s^−1^	35.1 ± 8.4	32.6 ± 12.3		30.5 ± 12.1		25.5 ± 6.4		26.6 ± 6.9	
VAP	µm s^−1^	57.8 ± 13.4	42.6 ± 12.6		39.2 ± 11.1		35.2 ± 6.1		35.5 ± 7.5	

Total motility (TM), progressive motility (PM), curvilinear velocity (VCL), straight-line velocity (VSL), average path velocity (VAP)

## HT analysis

**Fig. 3:**
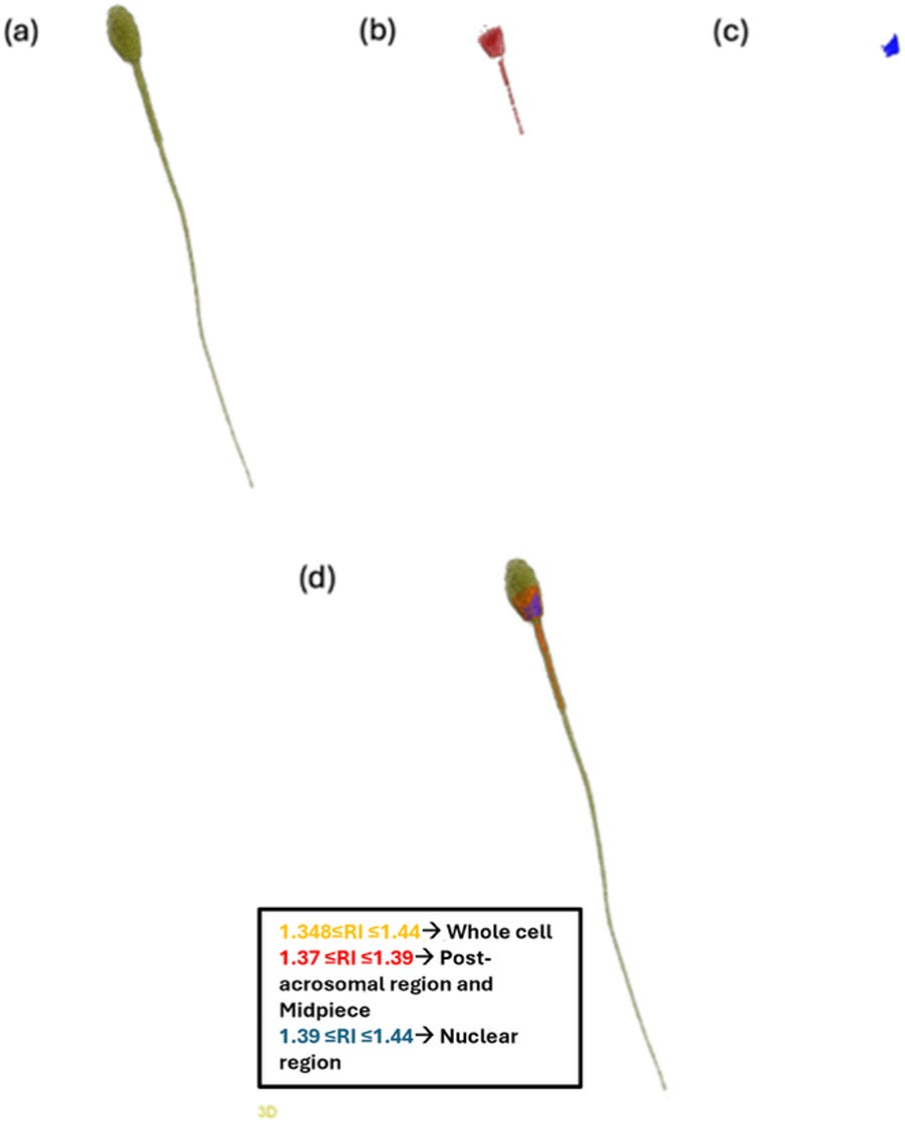
Holographic Tomographic Analysis of Stallion Spermatozoon Substructures Based on Refractive Index Values. (**a**) Whole cell, (**b**) post-acrosomal region and midpiece, (**c**) nuclear region and (**d**) merged tomogram with subcellular structures corresponding to different RI ranges reported with different colors.

HT analysis was performed to obtain 3D RI tomograms of equine spermatozoa, allowing quantitative assessment of subcellular regions, including the post-acrosomal region and midpiece, the nuclear region, and the whole cell. RI ranges adapted from Kim et al.^23^. were assigned to each structure, assuming compositional similarity across species:

- 1.348–1.44: whole sperm structure (Fig. 3a);
- 1.37–1.39: post-acrosomal region and midpiece (Fig. 3b);
- 1.39–1.44: nuclear region (Fig. 3c).

An example of an RI tomogram of a fresh spermatozoon is shown in Fig. 3d), with structures color-coded by RI range. The same RI intervals have been applied to all examined sperm cells, including both refrigerated and frozen samples preserved with different extenders. Holograms were processed to extract the structural parameters, enabling quantitative comparisons across structural regions and sample conditions.

Results highlighted that cryopreservation led to a general reduction in sperm volume, surface area, and dry mass compared to refrigeration, likely due to acrosomal reactions triggered by freezing^24^. However, volume was found to be the TomoAnalysis parameter most influenced by sperm freezing; thus, only the results related to this parameter are reported here.

**Fig. 4:**
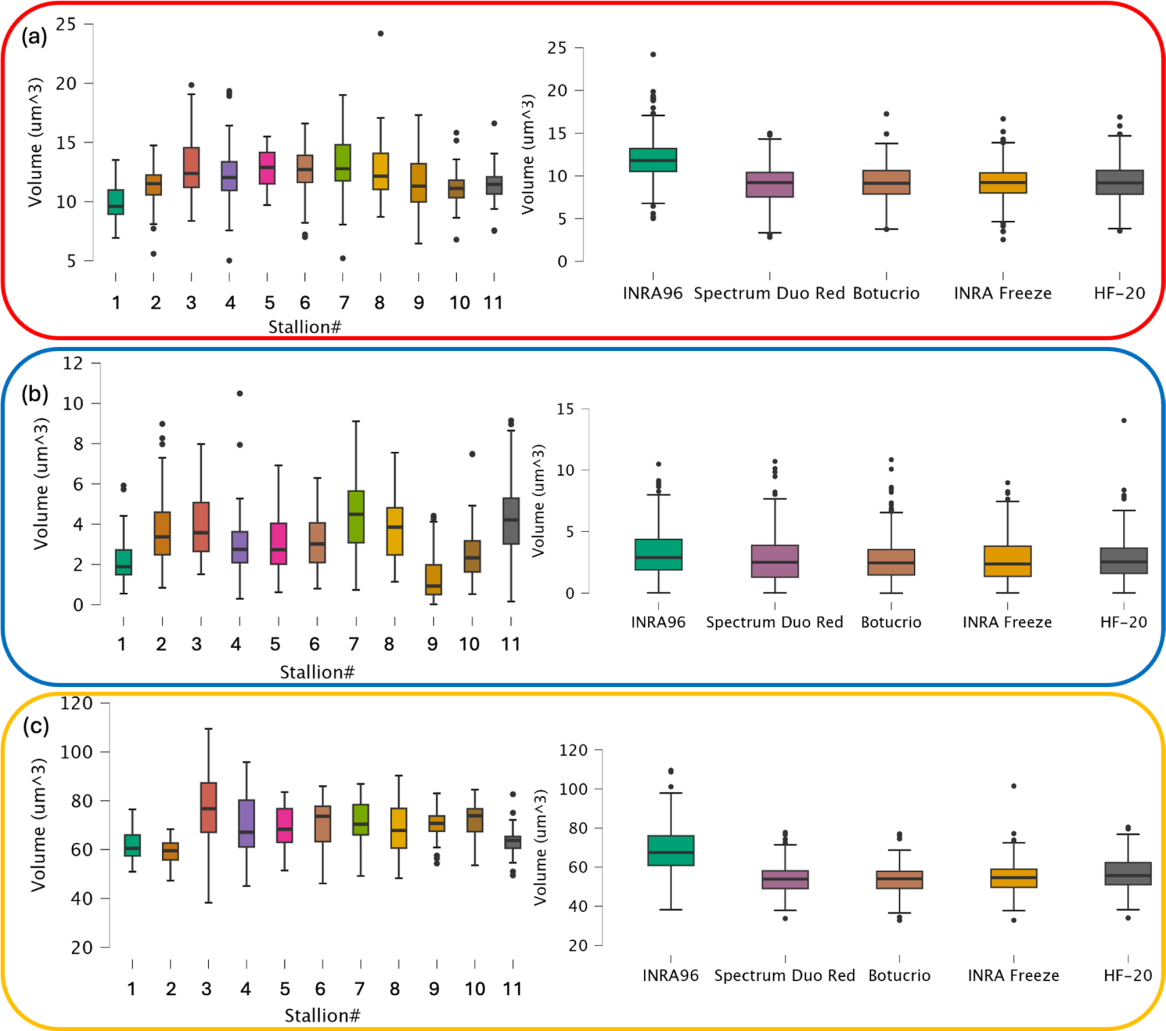
Sperm volume assessment. Sperm volume assessment conducted for three analyzed regions corresponding to different refractive index ranges: (**a**) Post-acrosomal region and Midpiece, (**b**) Nuclear region, and (**c**) Whole cell. The box plots on the left illustrate individual variations in fresh stallion sperm, while those on the right depict extender-related variations in fresh and frozen/thawed sperm, independent of individual variation.

The box plots in Fig. 4 illustrate the distribution of volume measurements for the three studied regions - mid-piece (Fig. 4a), nuclear region (Fig. 4b) and whole cell (Fig. 4c) - corresponding to different refractive index ranges (tables corresponding to the box plots are reported in Figure S2). These results were analyzed for fresh and frozen spermatozoa across different donors, regardless of the freezing extender used (box plots on the left side of Fig. 4) and for all donors under varying freezing extenders (box plots on the right side of Fig. 4).

The volume reduction observed after cryopreservation was particularly evident in the post-acrosomal and midpiece regions. Among the different freezing extenders, Spectrum Duo Red exhibited the most pronounced impact, with volume reductions exceeding 50% in some cases, whereas INRA Freeze showed lower reductions (Fig. 4a). In contrast, the nuclear region remained relatively stable, with only marginal optical density changes, in fresh and frozen sperm samples (Fig. 4b).

Statistical analysis confirmed significant differences (*p* < 0.001) in sperm volume across all three regions between fresh and frozen sperm with all four freezing extenders (Figure S3).

To further assess the ability of stallion spermatozoa to maintain structural integrity after cryopreservation, we related HT sperm traits after freezing with different extenders with the corresponding measurements in fresh semen, introducing HT Freezability Index (*FI*_*HT*_). This parameter is defined as the ratio between the volume measured for the three identified sperm regions - midpiece, nuclear region, and whole cell - across the respective RI ranges measured for each of the four freezing extenders, and the volume of the same regions in fresh sperm using INRA 96 as extender:

1 $$FI_{{HT}} = \frac{{V_{{freezing~extender}} }}{{V_{{INRA~96 }} }}*100$$

An *FI*_*HT*_ value of 100% indicates that no structural changes occurred due to freezing, suggesting optimal preservation. Values below 100% reflect a reduction in volume post-freezing, possibly due to dehydration or structural collapse. Conversely, values above 100% suggest an increase in volume, which may indicate swelling or other structural alterations. Comparing *FI*_*HT*_ values across different donors allows the assessment of inter-individual variability in sperm cryotolerance and identify stallions with superior freezability potential.

**Fig. 5:**
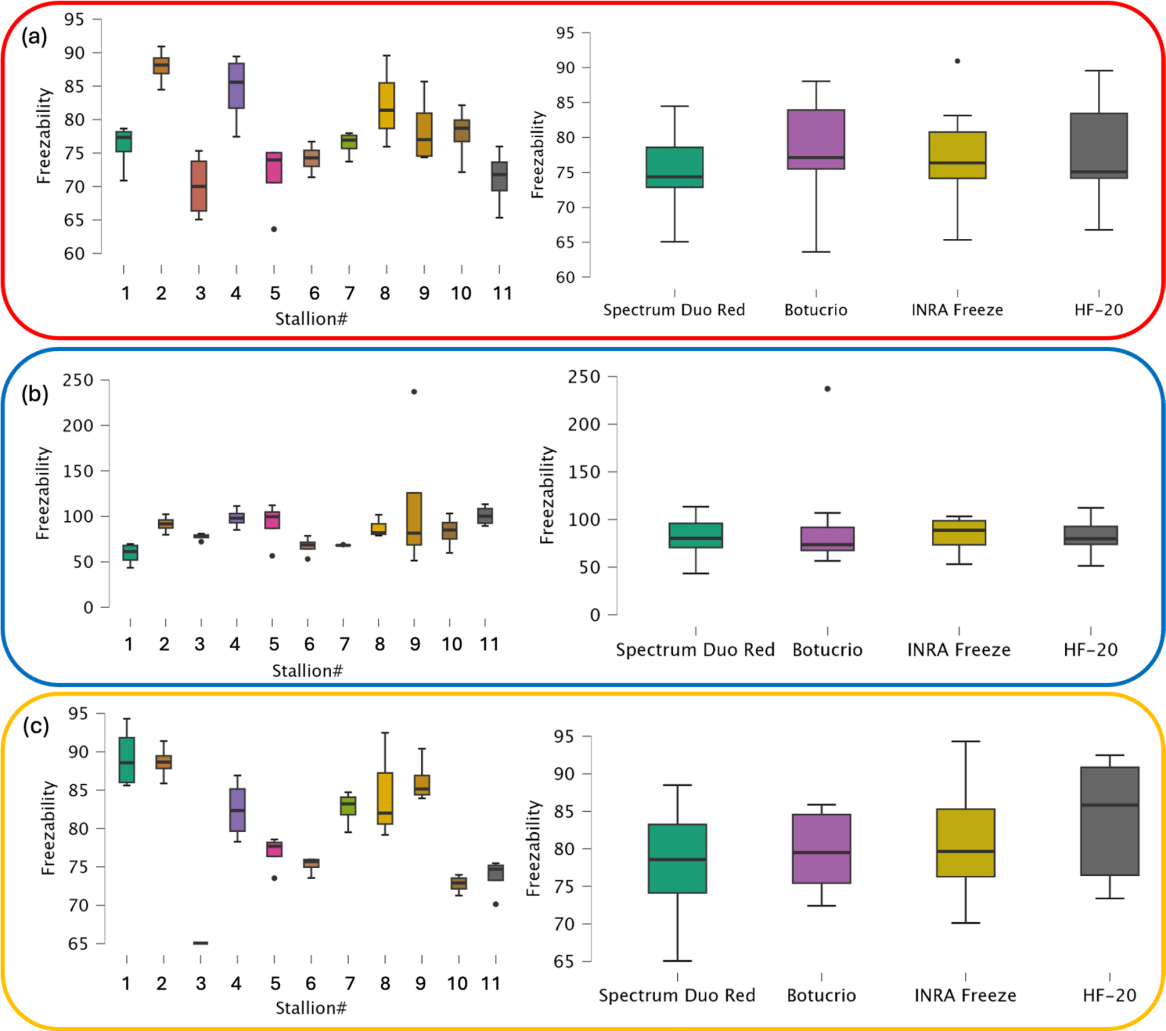
Distribution of HT Freezability index. *FI*_*HT*_ evaluated for the three analyzed regions corresponding to different refractive index ranges: (**a**) Post-acrosomal region and Midpiece; (**b**) Nuclear region; (**c**) Whole cell. Box plots on the left side represent the analysis across different donors. Box plots on the right side represent the results obtained for all donors under different freezing extenders, compared to the fresh sperm extender (INRA 96).

The Fig. 5 presents the HT Freezability values for different stallion donors across the three studied sperm regions: post-acrosomal region and midpiece (Fig. 5a), nuclear region (Fig. 5b) and whole cell (Fig. 5c). The left panels display the inter-individual variability in freezability, highlighting differences in sperm structural response to cryopreservation. The right panels compare *FI*_*HT*_ values for all donors under different freezing extenders (Spectrum Duo Red, BotuCrio, INRA Freeze, and HF-20), using INRA 96 as the fresh sperm extender. Tables corresponding to the presented box plots are reported in Figure S4.

HT data were correlated with both sperm kinetics and age-related parameters from different stallions. A good correlation with age was observed only for BotuCrio when whole cell volume is considered (*R*=−0.803, *p* = 0.009), whereas correlations with some kinetic parameters were found, as reported in the supplementary Table S1. However, no clear overall relationship emerged.

## Discussion

Analysis of sperm kinetics suggests that spermatozoa from different stallions respond differently to freezing, regardless of the extender used. Although the four extenders - three commercial products and one semi-defined extender prepared in our laboratory - differed in freezing protocols, equilibration times, and cooling rates, all led to a significant reduction in sperm motility (freezability index, see Figure S1) compared to fresh semen, with no substantial differences among the extenders. These findings emphasize the impact of intrinsic donor characteristics on sperm cryotolerance, reinforcing the necessity of personalized cryopreservation strategies to optimize post-thaw sperm functionality.

HT analysis confirmed that fresh sperm showed a higher and more uniform volume in the post-acrosomal region and midpiece than frozen sperm. Since this sperm region contains mitochondrial, which supply energy for movement through ATP production via oxidative phosphorylation following glycolysis^25,26^, this finding may be associated with the higher metabolic activity and motility typically observed in fresh sperm. A higher variability in post-acrosomal region and midpiece volume was observed among the freezing extenders (Fig. 4a), likely due to the impact of cryopreservation procedures and extenders on cellular integrity. In contrast, the nuclear region (Fig. 4b) displayed only minor changes. However, statistical analysis of the nuclear region across all extenders (Figure S3) revealed that fresh sperm maintained a more stable and preserved nuclear volume compared to frozen samples, which exhibited greater variability. This suggests that the freezing-induced injuries potentially affecting chromatin stability vary among freezing extenders with possibly stallion-freezing extender interaction^6^. When the whole cell is considered, ANOVA analysis confirmed significant volume differences among extenders, emphasizing the broad impact of cryopreservation media on overall sperm structure. Once again, fresh sperm (INRA 96) exhibited significantly better volume retention than frozen sperm, regardless of the freezing extender, aligning with the findings from the other sperm regions analyzed by HT. Among the freezing extenders, INRA Freeze and HF-20 appeared to retain volume more effectively than BotuCrio and Spectrum Duo Red, suggesting differences in their cryoprotective efficiency. This may be attributed to differences in the cooling rates during the equilibrium period: Spectrum Duo Red and BotuCrio undergo rapid freezing, while HF-20 and INRA Freeze cool more slowly. Rapid freezing procedure can induce higher osmotic stress^10^, leading to greater volume loss. However, based on the results reported in Figure S3, these differences were statistically relevant (*p* < 0.001) only for HF-20 when the whole cell is considered. In the sperm analysis conducted exclusively on fresh semen (INRA96), the differences between stallions were highly significant (data not shown), indicating a strong and statistically significant donor-dependent variability, while extender-dependent variability was comparatively lower. These results align with the findings presented in Fig. 4 (left panels), suggesting that the observed variations in the three HT regions are more likely attributable to individual donor differences rather than extender effects. This supports previous observations by other researchers^27,28^ regarding the high individual variability of equine semen.

Regarding the HT Freezability Index, results reported in Fig. 5 highlight that, overall, most stallions exhibit *FI*_*HT*_ values below 100%, indicating a reduction in sperm volume post-freezing, according with the boxplot reported in Fig. 4. This decrease is likely due to dehydration and structural contraction and can be linked to the lower metabolic activity and motility typically observed in frozen sperm respect to fresh sperm. The midpiece and post-acrosomal region (Fig. 5(a) and S4(a)) appears to be the most affected, with several donors displaying *FI*_*HT*_ values significantly below 80%, suggesting substantial structural shrinkage. In contrast, the nuclear region and the whole cell (Figs. 5(b), 4(c), S4(b), S4(c)) show a lower response, with *FI*_*HT*_ values higher than to 80% across most donors. Variability among donors is evident, with some individuals showing a greater tolerance to cryopreservation. Notably, stallions #2 and #4, with *FI*_*HT*_ values near to 100% for all the three considered regions, displayed greater resilience to cryopreservation, highlighting potential “good cooler” phenotypes. Classifying donors as “good " or “bad” coolers provides valuable insights into individual variability in cryopreservation resilience^28–30^ and supports the development of optimized preservation protocols tailored to donor-specific needs.

Among the freezing extenders, Spectrum Duo Red tended to induce lower *FI*_*HT*_ values, especially in the post-acrosomal and midpiece region, indicating a greater reduction in volume post-freezing. Conversely, INRA Freeze, BotuCrio and HF-20 better preserved sperm structure, as indicated by higher *FI* values closer to 100% across all analyzed regions. Although no statistically significant differences were detected (*p* > 0.1, see Tables S5), the observed trends suggest potential biological effects that may require further investigation with a larger sample size or different experimental conditions. These findings suggest that both donor-specific factors and extender choice play a crucial role in maintaining sperm integrity after cryopreservation, as also highlighted in Figure S6, where an inhomogeneous response to the four extenders is clearly visible for each donor.

## Conclusion

This study highlights significant variability in sperm structural integrity following cryopreservation, influenced by both the type of cryopreservation medium and donor-specific factors. Our findings demonstrate that holographic tomography is an effective tool for evaluating the impact of cryopreservation on sperm integrity, providing a robust framework for future research.

The observed inter-individual variability in sperm responses to cryopreservation underscores the importance of considering donor-specific factors, such as membrane composition, intracellular water content, and osmotic stress tolerance. This variability was particularly evident in the differences in the volumetric ratios of acrosomal, nuclear, and whole-cell regions, suggesting that some donors exhibit better structural preservation than others.

Additionally, comparison of freezing extenders revealed that certain media were more effective in maintaining nuclear and midpiece integrity across multiple donors, suggesting that extender choice should be tailored to individual sperm characteristics. In particular, cryopreservation media exhibited varying degrees of volume reduction, with INRA Freeze and HF-20 offering a slightly better preservation compared to BotuCrio and Spectrum Duo Red.

These results emphasize the necessity of personalized approaches in developing cryopreservation protocols, with a focus on optimizing preservation strategies for different donor profiles. Future studies should further explore genetic or biochemical factors influencing donor-specific freezing tolerance and examine correlations between structural integrity, post-thaw motility, and fertilization capacity to refine sperm preservation techniques.

## Materials and methods

### Reagents

All reagents and media were purchased from Merck (Milan, Italy) unless otherwise stated and cell culture tested.

### Animal collection and breeding care

Between April and May 2024, a single ejaculate was collected from each of eleven stallions used for semen production. These stallions, ranging in age from 10 to 20 years, represented different breeds and had proven fertility. All were clinically healthy and fed a standard diet of mixed hay and basic concentrate, without antioxidant supplements, with unlimited access to water. The stallions were housed individually in paddocks at the Regional Center of Equine Improvement (Centro Regionale di Incremento Ippico - S. Maria Capua Vetere, Caserta). This facility is authorized by the Regional Government of Campania, Italy, for equine semen collection (authorization number: U 1500083 CE000642004) and adheres to strict health and animal welfare standards.

All animal procedures adhered to the European Union guidelines (Directive 2010/63/EU and D. Lgs. 4/03/2014 n. 26) and followed the ARRIVE guidelines, which emphasize minimizing the number of animals used and reducing any pain or stress. Since the experimental design does not fall under the scope of European Directive 63/2010, the Ethics Committee of the University of Basilicata (Organismo per il Benessere Animale, OpBA) determined that no specific authorization was needed for this study (Protocol code: OpBA 14_2024_UNIBAS, approved on 11 July 2024). The fertility of all stallions had been previously confirmed, as evidenced by the production of viable offspring obtained through artificial insemination with semen collected during routine sampling.

### Sperm collection, Dilution and shipping

Semen was collected using a Missouri artificial vagina equipped with an in-line sterile gauze to filter out the sperm gel fraction. Prior to sampling, each stallion underwent at least three preliminary semen collections, conducted twice weekly, to empty the epididymal sperm reservoir. The semen was divided into four aliquots and diluted at a 1:2 ratio (semen: extender) with INRA 96 (INRA 96, IMV Technologies, L’Aigle Cedex, France). This extender was chosen because it is one of the most widely used extenders in the literature for horse semen preservation and demonstrated an excellent ability to store both equine and donkey semen at refrigeration temperatures^11,31^. INRA 96 aliquots were stored in sterile tubes at + 4 °C and pre-warmed to 37 °C before semen collection. After adding the semen, the tubes were placed in a jar containing one liter of water at 37 °C, which was housed in a polystyrene box with frozen cooling tiles. This setup gradually lowered the sample temperature to room temperature (+ 20 °C) over approximately two hours, with a cooling rate of about 0.10–0.15 °C/min.

### Sperm concentration and kinetics

Upon arrival at the Laboratory of Biology and Technology of Animal Reproduction at the University of Basilicata, Potenza, semen samples were evaluated for sperm concentration and kinetics. This was done using a Makler chamber (Sefi-Medical Instruments, Haifa, Israel) and computer-assisted sperm analysis (SCA 5.0 system, Microptic, Barcelona, Spain). After measuring sperm concentration, each sample was diluted with INRA 96 to reach a concentration of 30 × 10^6^ sperm mL^−1^ for SCA analysis. The samples were then equilibrated for 2 min at 37 °C on a heated microscope stage. Spermatozoa with an average velocity below 10 μm s^−1^ were classified as immotile. The sperm kinetics parameters measured included: (i) the percentage of motile sperm (TM); (ii) the percentage of progressively motile sperm (PM), defined as those with an average path velocity exceeding 30 μm s^−1^ and track straightness above 80%; (iii) curvilinear velocity (VCL, µm s^−1^); (iv) straight-line velocity (VSL, µm s^−1^); and (v) average path velocity (VAP, µm s^−1^). For each sample, the tracks of at least 1,000 spermatozoa were recorded and analyzed in duplicate, with each batch containing more than 500 spermatozoa.

### Sperm freezing

The semen samples were centrifuged at 300 x g for 10 min and then, resuspended in one of the four considered freezing extenders used in this research to reach a final sperm concentration of 100 × 10^6^ sperm mL^−1^. These samples were subsequently incubated under specific conditions of time and temperature (detailed below) to allow for diffusion and equilibration of the cryoprotective agents (CPAs) between the extracellular and intracellular compartments. Equilibration times were determined based on either literature recommendations or manufacturer instructions, as outlined below. The four freezing extenders used in this study are known for their effectiveness in stallion sperm cryopreservation. Three of them are commercial products with proprietary compositions, while HF-20 is a semi-defined medium produced in our laboratory with the composition listed below.

1. Spectrum duo red: equilibration time = 5 min at room temperature. After equilibration, the semen doses were placed at −20 °C for 3.5 min and then exposed to liquid nitrogen (LN_2_) vapors.
2. BotuCrio: equilibration time = 20–30 min at + 4 °C. Semen doses were placed in a refrigerator at + 4 °C for 20–30 min and then exposed to LN_2_ vapors.
3. INRA freeze: equilibration time = 90 min with a decreasing temperature gradient down to + 4 °C. The tubes with semen were initially dipped in a bucket of water at + 20 °C and then placed in a refrigerator set at + 4 °C for 60 min. Afterward, these tubes were transferred to a cold handling cabinet at + 4 °C, and the sperm doses were packaged in straws. The sperm doses were then placed in a + 4 °C refrigerator for an additional 15 min before exposure to LN_2_ vapors.
4. HF-20^11,32^: equilibration time = 90 min with a decreasing temperature gradient down to + 4 °C. HF-20 contains 5 g glucose, 0.3 g lactose, 0.3 g raffinose, 0.15 g sodium citrate, 0.05 g sodium phosphate, 0.05 g potassium sodium tartrate, 10% egg yolk (EY), 25,000 IU penicillin, 0.08 g streptomycin, 3% glycerol, and ultrapure water up to 100 mL. The procedure for packaging and freezing the semen doses was the same as that described above for INRA Freeze.

### Sperm freezing and thawing

Semen doses were prepared using individually labeled 0.5 mL polyvinyl chloride straws (IMV-Technologies, L’Aigle, France), which had been pre-cooled to + 4 °C. After filling the straws with semen, they were sealed using a filling powder (Poudre de bouchage bleue, IMV-Technologies) and arranged horizontally in a freezing rack (distribution block for 40 medium straws, IMV-Technologies). At the time of freezing, the rack was placed inside a polystyrene box containing LN_2_, positioning the straws 4 cm above the LN_2_ surface for 10 min before plunging them into the LN_2_ for storage. One week after freezing, four straws from each extender for each stallion were thawed in a water bath at 37 °C for 30 s prior to semen analysis. Following the initial centrifugation to remove the freezing extender, the sperm pellet was resuspended in 400 µL of INRA 96. The samples were then placed on a discontinuous Percoll gradient (70/40)^33^ and centrifuged at 1000 x g for 15 min to remove EY residues, which could interfere with subsequent sperm analyses. The resulting pellet was divided in two aliquots. One of them was resuspended in INRA 96 and examined under a microscope for sperm motility and kinetics. The other one was resuspended in PBS-PVA and fixed with paraformaldehyde (PFA) for 1 h at 4 °C. Fixed samples were, then, centrifuged to remove the PFA and resuspended in PBS-PVA supplemented with 0.1% sodium azide and stored at 4 °C for some weeks for holographic tomography assessment.

### Holographic tomography (HT)

HT enables the creation of a 3D RI map of the sample, which is invaluable for the detailed characterization of biological specimens or transparent materials.

In this study, the HT-2H commercial system from Tomocube was used. This holotomographic microscope is a holistic imaging tool; it captures high-resolution 3D images without the need for labels or staining, allowing the observation of cell morphology dynamics in real-time, with capabilities for long-term imaging. HT-2H system employs Mach-Zehnder interferometry with a 532 nm LED laser, splitting the beam into sample and reference paths. The sample is illuminated and observed through a 60x high-NA water immersion objective, capturing multiple 2D holograms at various incident angles using a digital micromirror device (DMD) for 3D refractive index mapping. It provides high lateral (110 nm) and axial (220 nm) resolution, making it ideal for in-depth biological research^34,35^. Each examined sample was holographically analyzed to compare fresh and frozen samples and to assess any changes after freezing. An aliquot of 60 µL of each analyzed sample were placed on a glass Tomodish substrate and observed without staining using the Tomocube HT-2H optical diffraction tomography system. Holograms of 40 spermatozoa were acquired for each sample. TomoStudio software has been used to process the holograms and to create detailed cell morphologies, while TomoAnalysis software allowed to apply deep learning techniques for segmentation and quantitative analysis. Structural parameters, including refractive index (a.u.), volume (µm^3^), surface area (µm^2^), sphericity (a.u.), projection area (µm^2^), dry mass (pg), and concentration (pg/µm^3^), were obtained.

### Statistical analysis

The statistical analysis of the data was performed using ANOVA (Analysis of Variance), a robust method for evaluating differences among multiple groups. This technique allowed for the identification of statistically significant variations within the datasets, ensuring a reliable comparison of experimental conditions. By assessing variance both within and between groups, ANOVA provided valuable insights into the significance of the observed differences, contributing to the overall reliability and accuracy of the study’s findings. Before the analyses, percentage values were transformed in arcsine. Normal data distribution and homogeneity of variance were assessed by the Shapiro–Wilks test and Levene’s test, respectively. Pair-wise comparisons of the means were performed with the Bonferroni test. The threshold of *p* < 0.05 was used as the minimum level of statistical significance. The analysis was performed by using JASP, an open-source statistics program that is free, friendly and flexible^36^.

## Supplementary Information

Below is the link to the electronic supplementary material.

Supplementary Material 1

## Data Availability

The datasets generated during and/or analysed during the current study are available from the corresponding author on reasonable request.
