# Supplementary material for: Quantitative holographic analysis in stallion spermatozoa following cryopreservation
Source: Sci Rep. 2025 Dec 4;15:43190. doi: 10.1038/s41598-025-24885-w (PMC12680650; doi:10.1038/s41598-025-24885-w)
Supplement: Supplementary file 1 — Supplementary Material 1 [file 41598_2025_24885_MOESM1_ESM.docx]

**SUPPLEMENTARY MATERIALS**

**Quantitative Holographic Analysis in Stallion Spermatozoa following cryopreservation**

*Graziano Preziosi^1^, Raffaele Boni^2^, Raffaella Ruggiero^2^, Stefano Cecchini Gualandi^2^ and Maria Antonietta Ferrara^1^**

1. Institute of Applied Sciences and Intelligent Systems, Unit of Naples, Italian National Research Council (ISASI-CNR), Via Pietro Castellino 111, 80131 Napoli, Italy
2. Department of Basic and Applied Sciences (DiSBA), University of Basilicata, Via dell’Ateneo Lucano, 10, 85100 Potenza, Italy

* Correspondence: antonella.ferrara@na.isasi.cnr.it


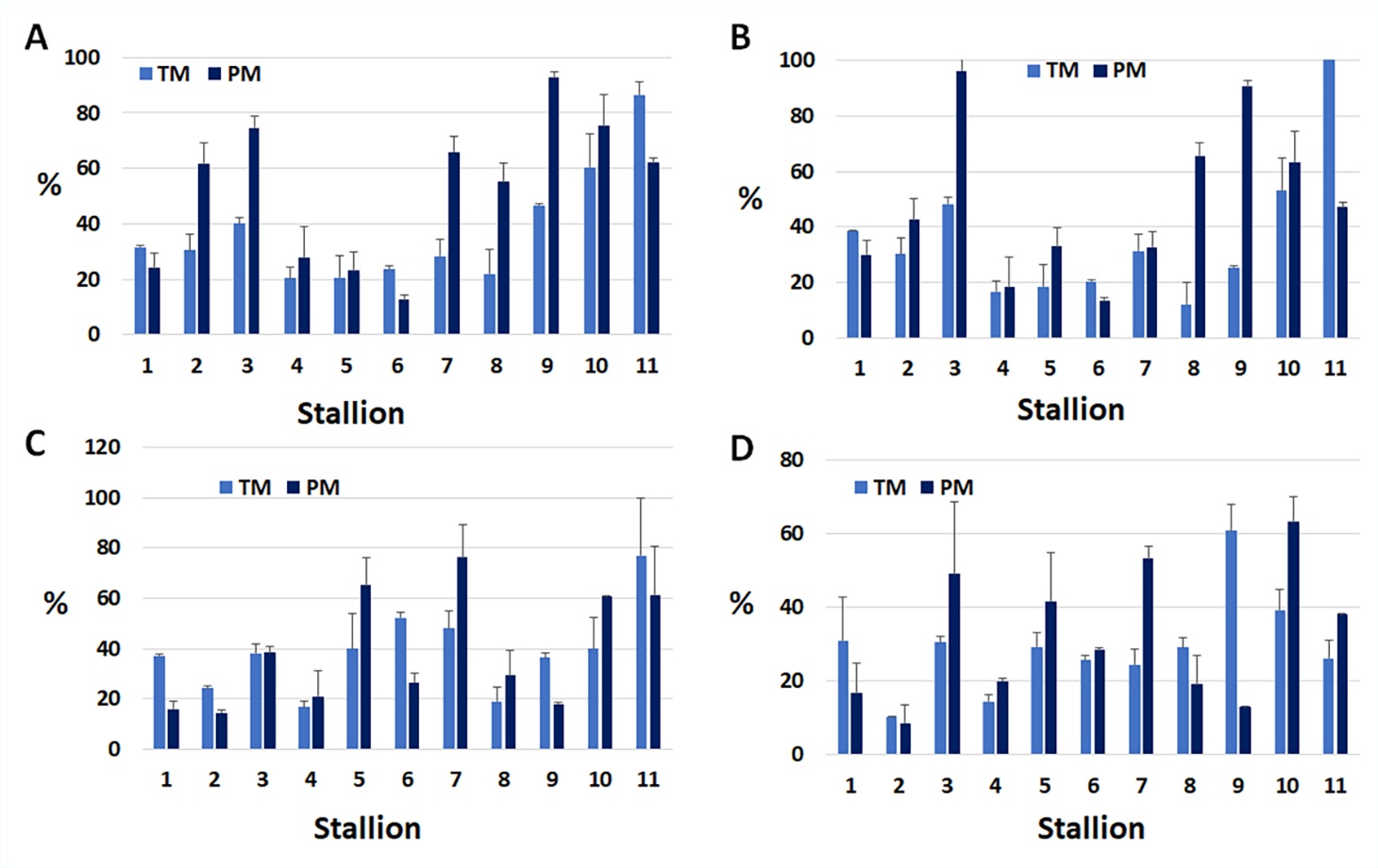


**Figure S1. Comparison of Stallion Sperm Freezability Using Different Cryopreservation Extenders.** The stallion sperm freezability index (mean ± SD) was evaluated by comparing total motility (TM) and progressive motility (PM) after and before freezing. Each panel illustrates the results of freezing stallion ejaculates using Spectrum Duo Red (A), Botucrio (B), INRA Freeze (C), and HF-20 (D).


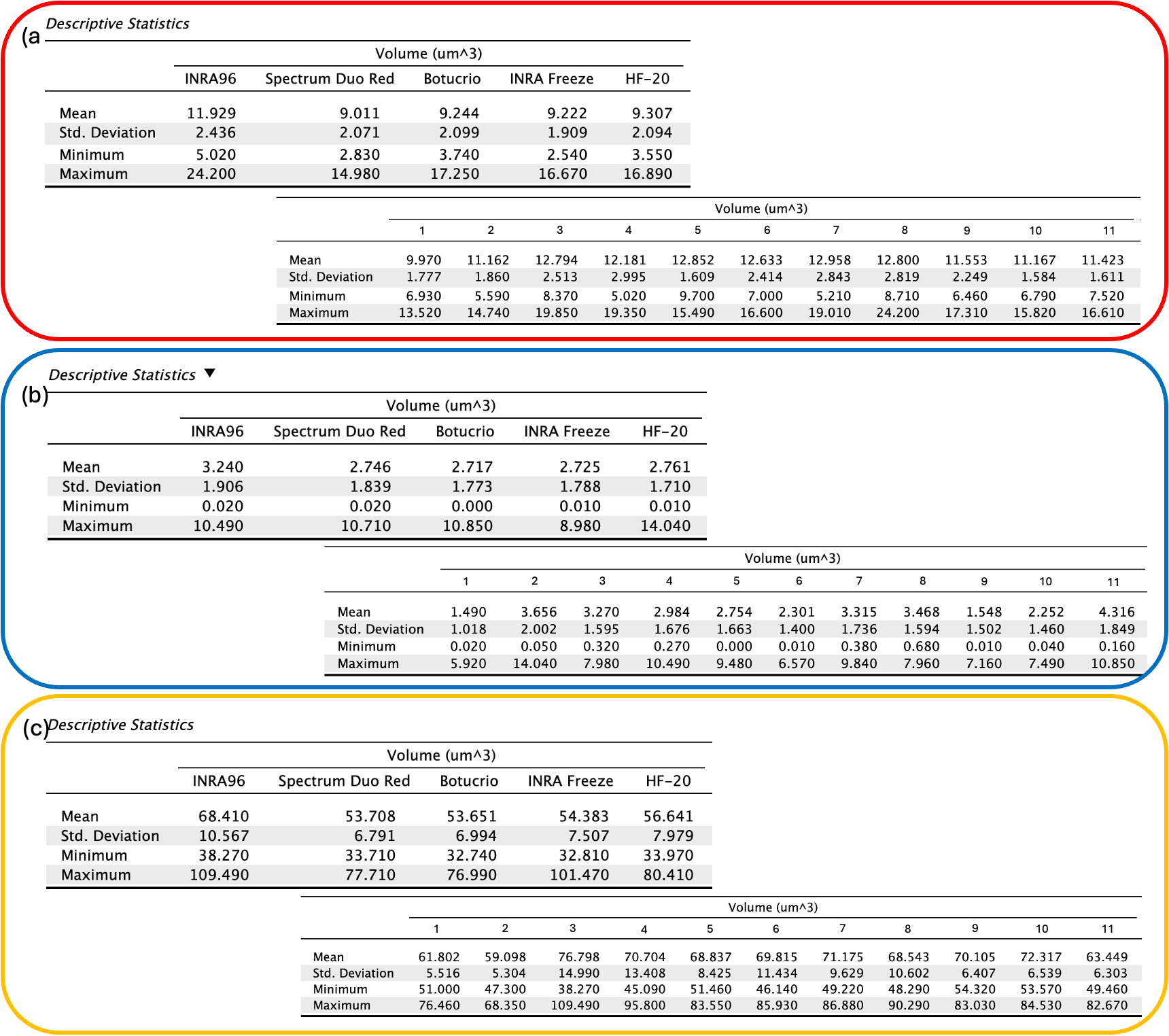


**Figure S2:** Mean, Std. Deviation, Minimum and Maximum values of volume measurements for the three analyzed regions corresponding to different refractive index ranges: (a) Mid piece; (b) Nuclear region; (c) Whole cell. Tables on the right side represent the results analyzed using the INRA 96 extender for fresh semen across different donors. Tables on the left side represent the results obtained for all donors under different extenders: INRA 96 for refrigeration and the four selected freezing extenders.


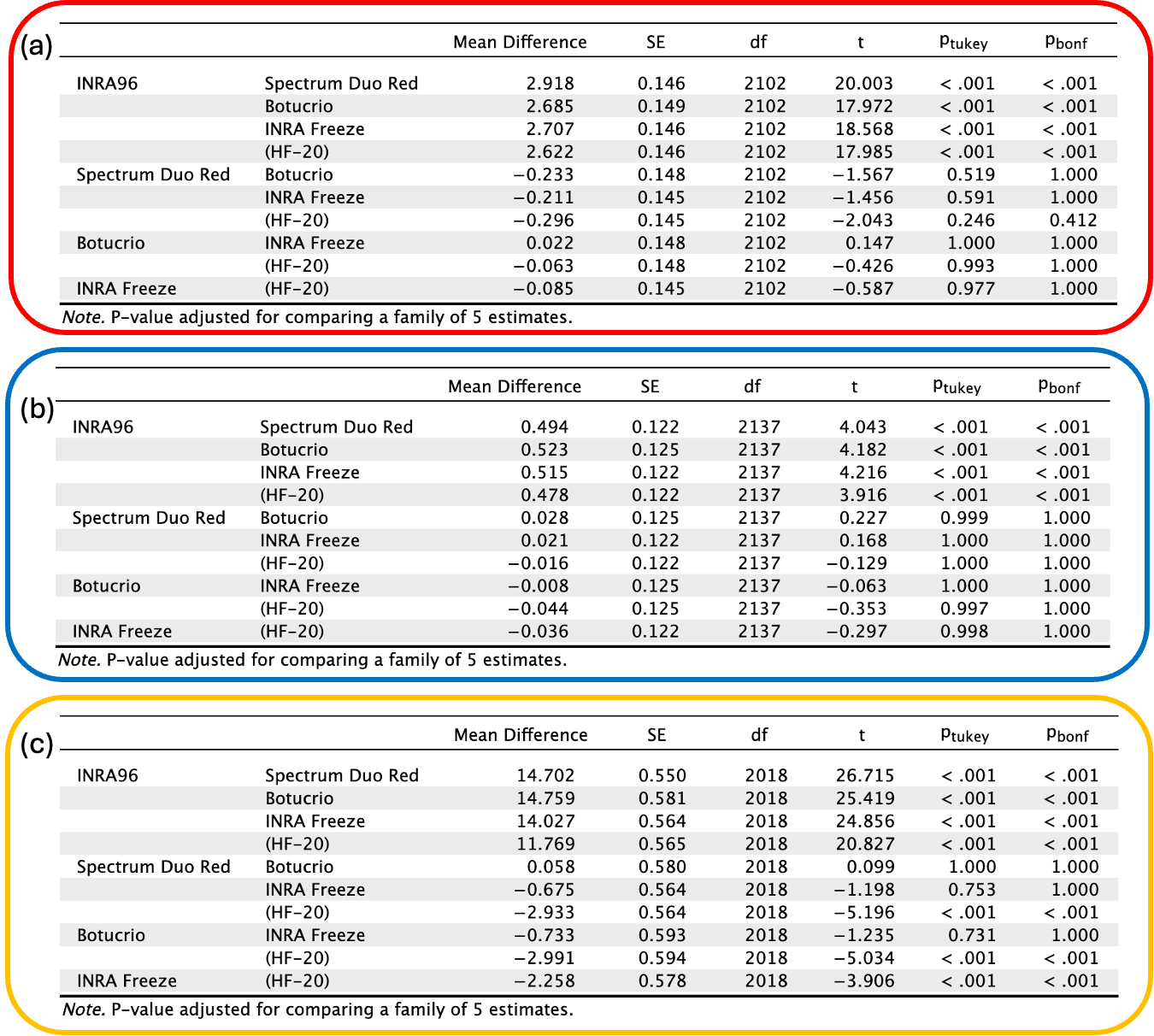


**Figure S3**: ANOVA test on volume over the different examined extenders obtained for the three analyzed regions corresponding to different refractive index ranges: (a) Mid piece; (b) Nuclear region; (c) Whole cell.


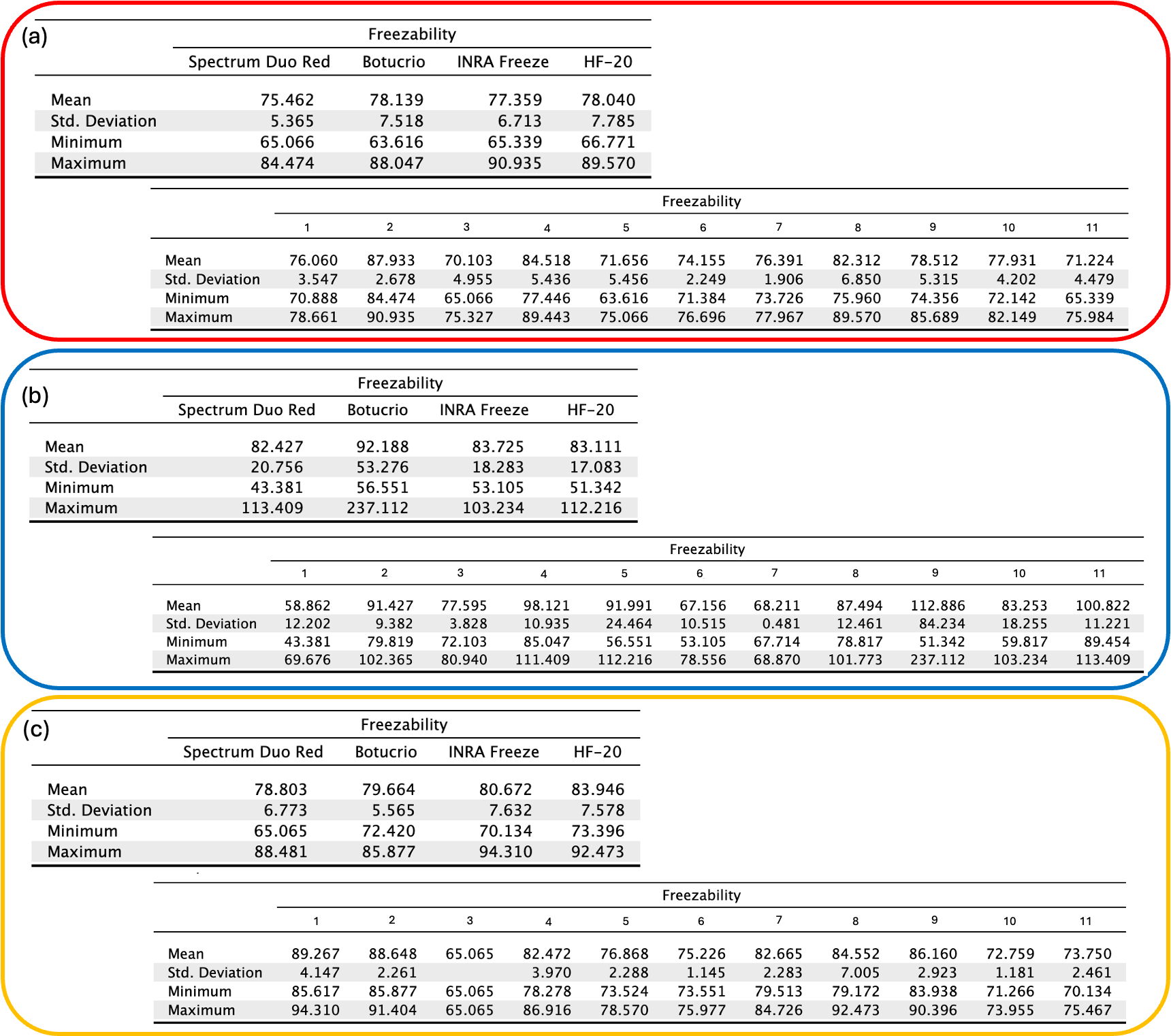


**Figure S4**: HT Freezability index Mean value, Std. Deviation, Minimum and Maximum values obtained for the three analyzed regions corresponding to different refractive index ranges: (a) Mid piece; (b) Nuclear region; (c) Whole cell. Tables on the right side represent the analysis across different donors. Tables on the left side represent the results obtained for all donors under different extenders.

**Table S1.** Pairwise correlation coefficients (R) between sperm kinematic variables and HT volume traits, analyzed in samples of either fresh sperm or frozen sperm preserved with four extenders (Spectrum Duo Red, Botucrio, INRA Freeze, and HF-20).

Total motility (TM), progressive motility (PM), curvilinear velocity (VCL), straight-line velocity (VSL), average path velocity (VAP). * (p≤ 0.05); ** (p≤ 0.01); ***(p≤ 0.001).

|  |  | **Fresh sperm** | **Frozen sperm** | | | |
| --- | --- | --- | --- | --- | --- | --- |
|  |  |  | **Spectrum Duo Red** | **Botucrio** | **INRA Freeze** | **HF-20** |
| **Whole cell** | TM | -0.308 | -0.444 | -0.585 | -0.474 | +0.193 |
|  | PM | -0.029 | +0.263 | +0.288 | -0.135 | -0.227 |
|  | VCL | +0.763** | +0.661* | +0.661* | -0.330 | -0.299 |
|  | VSL | +0.941*** | +0.626* | +0.678* | -0.250 | -0.191 |
|  | VAP | +0.920*** | +0.632* | +0.690* | -0.425 | -0.163 |
|  |  |  |  |  |  |  |
| **Post-Acrosomal** | TM | -0.502 | -0.440 | -0.407 | -0.718** | -0.508 |
| **& Middle-Piece** | PM | -0.439 | -0.095 | +0.053 | -0.090 | -0.346 |
|  | VCL | +0.506 | +0.448 | +0.311 | -0.027 | -0.034 |
|  | VSL | +0.456 | +0.361 | +0.263 | +0.435 | +0.449 |
|  | VAP | +0.560 | +0.385 | +0.274 | -0.043 | +0.222 |
|  |  |  |  |  |  |  |
| **Nuclear Region** | TM | -0.150 | +0.170 | +0.510 | -0.017 | -0.746** |
|  | PM | -0.259 | -0.186 | +0.276 | +0.289 | +0.108 |
|  | VCL | -0.432 | -0.245 | -0.240 | +0.608* | +0.356 |
|  | VSL | -0.147 | -0.204 | -0.044 | +0.574 | +0.622* |
|  | VAP | -0.242 | -0.210 | -0.126 | +0.564 | +0.407 |


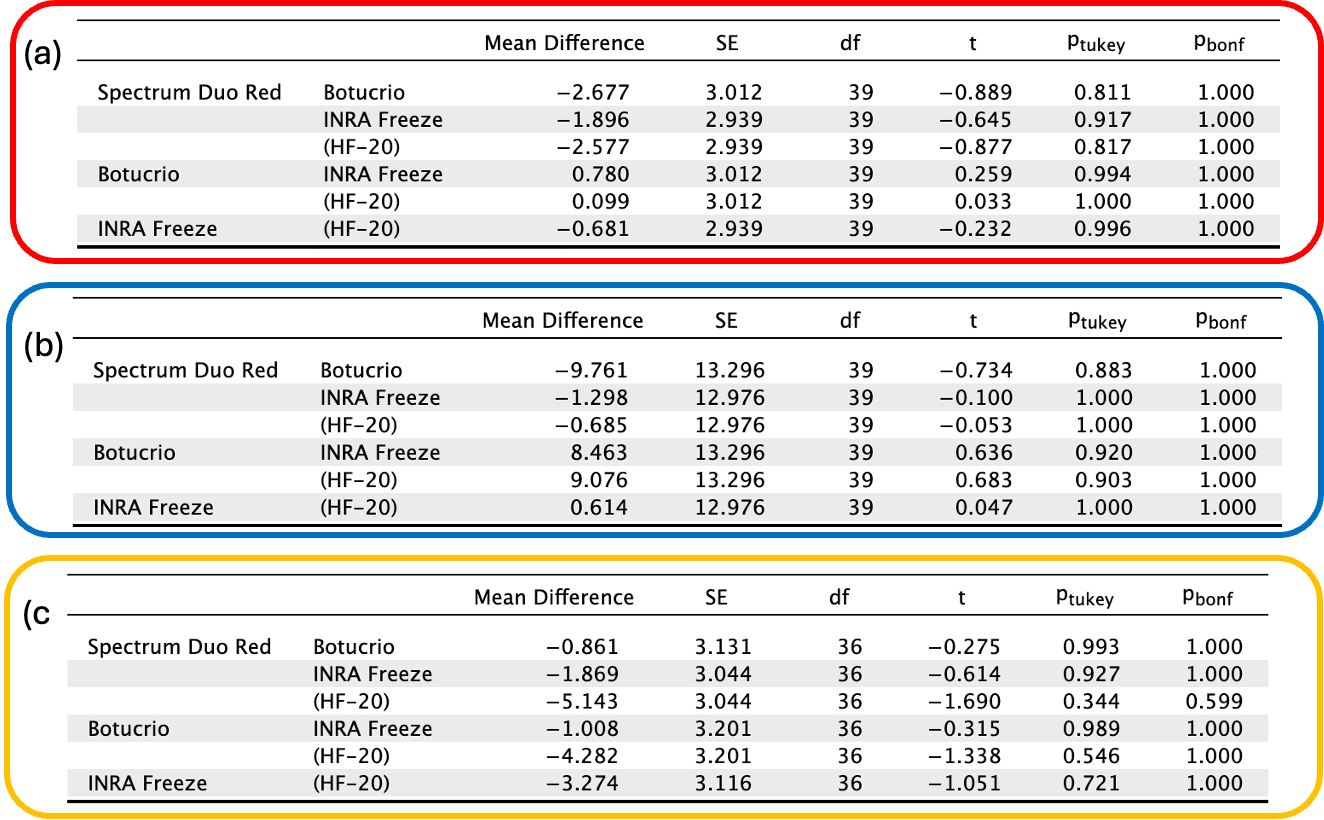


**Figure S5**: ANOVA test on HT freezability index over the different examined extenders obtained for the three analyzed regions corresponding to different refractive index ranges: (a) Mid piece; (b) Nuclear region; (c) Whole cell.
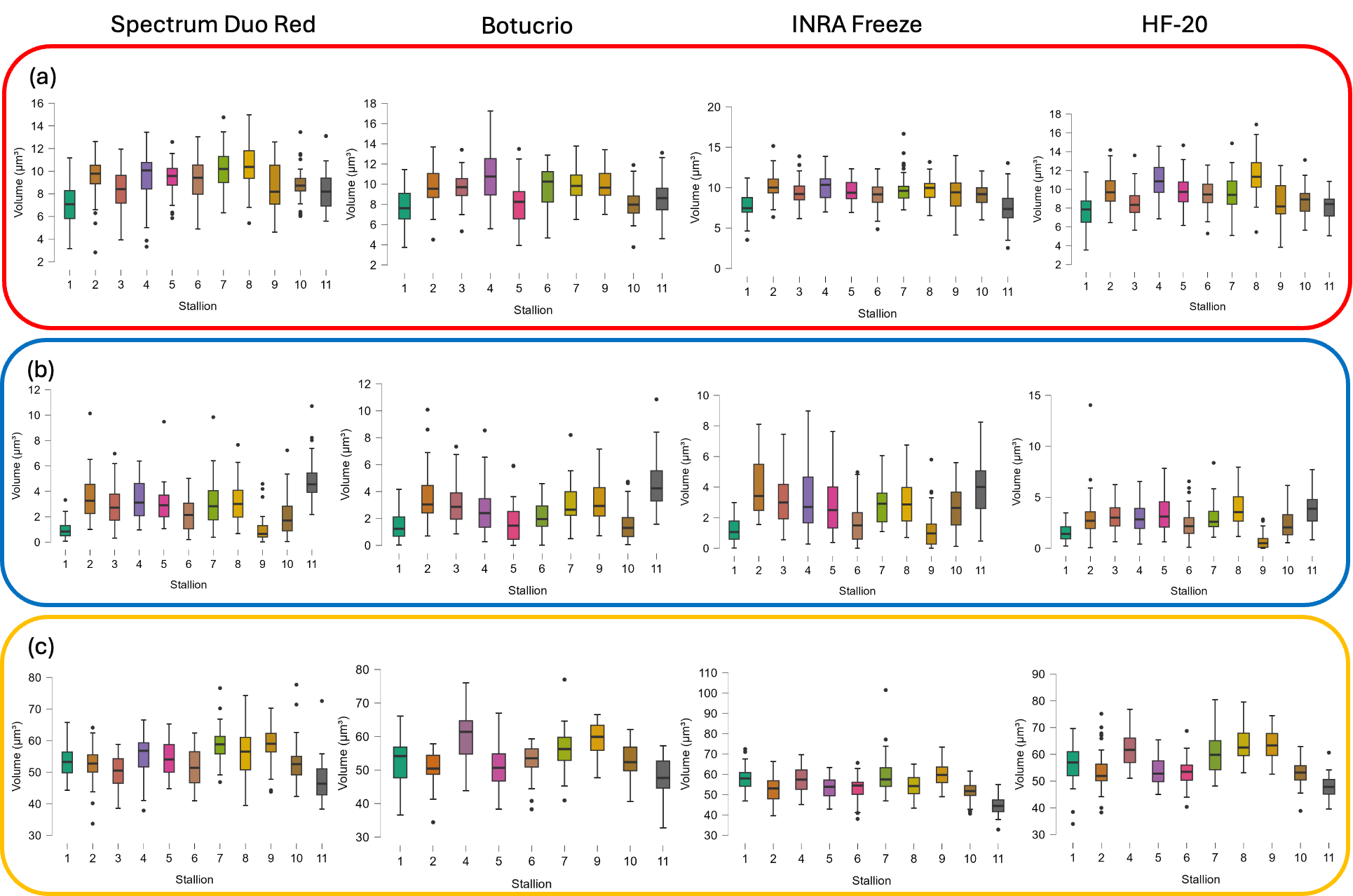


**Figure S6**: Volume of (a) post-acrosomal region and midpiece, (b) nuclear region, and (c) whole cell are reported for each stallion across the different extenders.
